# IGF-1 regulates the growth of fibroblasts and extracellular matrix deposition in pelvic organ prolapse

**DOI:** 10.1515/med-2020-0216

**Published:** 2020-09-02

**Authors:** Yitong Yin, Ying Han, Chang Shi, Zhijun Xia

**Affiliations:** Department of Obstetrics and Gynecology, Pelvic Floor Disease Diagnosis and Treatment Center, Shengjing Hospital of China Medical University, No. 36 San Hao Street, Heping District, Shenyang, 110004, China

**Keywords:** IGF-1, pelvic organ prolapse, human vaginal fibroblasts

## Abstract

This study was carried out to observe the impact of insulin-like growth factor-1 (IGF-1) on human vaginal fibroblasts (HVFs) in the context of pelvic organ prolapse (POP) and to explore its effects on mitogen-activated protein kinases (MAPK) and nuclear factor-κB (NF-κB) signaling pathways. First, it was found that IGF-1 expression reduced in the vaginal wall tissues derived from POP compared to that in non-POP cases. Then the role of IGF-1 was explored in HVFs and thiazolyl blue tetrazolium bromide (MTT) and flow cytometry were used to detect cell viability and cell apoptosis. Western blot assay and quantitative real-time polymerase chain reaction were used to detect the protein and mRNA expression. The results showed that knockdown of IGF-1 inhibited the cell viability of HVFs, promoted the cell apoptosis of HVFs, and decreased the expression of types I and III collagen in HVFs, which was through inhibiting the expression of IGF-1 receptor and MAPK/NF-κB pathways. However, IGF-1 plasmid had the opposite effects on HVFs. In conclusion, our results showed that IGF-1 could activate MAPK and NF-κB pathways, thereby enhancing collagen metabolism and the growth of vaginal wall fibroblasts then to inhibit POP development.

## 1 Introduction

Pelvic organ prolapse (POP) is characterized by the weakening of the pelvic floor support tissues, and it mainly affects the health of middle-aged and elderly women. In the next 30 years, the number of women over the age of 50 who need surgery for POP will increase by 45% [1]. And with the aging of society, 11–19% of women may need surgery for POP [2]. Although vaginal hysterectomy and anterior and posterior vaginal wall repair can improve the symptoms of vaginal prolapse in different degrees, it can distort or damage the normal anatomical relationship, and the recurrence rate is high.

The occurrence of POP is related to the destruction of the connective tissue structure and functional integrity of the pelvic floor [3,4]. The main constituent cells of the connective tissue are fibroblasts, which play key roles in maintaining the elasticity and toughness of connective tissue in the pelvic floor [5].

Collagen is an important member of the structural proteins in the extracellular matrix, it is synthesized and secreted by fibroblasts. Collagen is usually present in the form of insoluble fibers and is involved in maintaining tissue integrity. In addition, it can resist high-strength tension and is an important factor in determining the toughness of the connective tissues. The connective tissues at the pelvic floor mainly include types I (Col I) and III collagen (Col III) [6]. It is reported that the content of Col I and Col III in POP is reduced [7,8], and several studies [9,10,11] showed that increased collagen degradation is the main reason for the decrease in collagen content and POP.

Insulin-like growth factor (IGF) systems are a class of insulin-like polypeptides mainly synthesized by the liver, which are composed of IGFs and their receptors and IGF-binding proteins. IGFs regulate a variety of biological effects such as cell growth, metabolism, proliferation, and differentiation [12,13,14]. IGF-1 is a key factor in the insulin signaling pathway and a key growth factor related to various biological properties such as cell proliferation, differentiation, maturation, and survival [15]. Studies have shown that IGF-1 can inhibit collagen degradation and increase the number and activity of osteoblasts [16], so IGF-1 can promote osteoblast proliferation at a certain concentration range [17]. However, whether IGF-1 affects collagen in POP has not been reported.

Therefore, it was speculated that IGF-1 may affect the metabolism of collagen in POP and tried to explore the related mechanisms. In the present study, the effects of IGF-1 were investigated on the growth of vaginal wall fibroblasts and the metabolism of Col I and Col III and explored whether the effects were related to mitogen-activated protein kinases (MAPK) and nuclear factor-κB (NF-κB) pathway regulation.

## 2 Material and methods

### 2.1 Patient selection and tissue preparation

The tissues were obtained from the vaginal wall of 30 patients who had POP or those who are suffering from other diseases which also require hysterectomy at Shengjing Hospital of China Medical University. Informed consents were provided to all the subjects and then they signed the surgical consent forms before undergoing surgery. All the patients were evaluated clinically and used the Pelvic Organ Prolapse Quantification assessment to classify the POP stage [18]. The present study was approved by the Ethics Committee of Shengjing Hospital of China Medical University.

### 2.2 Culture and identification of the primary fibroblasts

From the Shengjing Hospital of China Medical University, human fibroblasts were obtained from the vaginal wall (HVFs) of five patients who had POP. Informed consents were provided by all the subjects and they signed the surgical consent forms before surgery. Briefly, phosphate buffer saline (PBS) (containing 1% amphotericin B, streptomycin, and penicillin) was used to wash the fresh vaginal wall tissues derived from the surgical margin of the free womb for three times at 4°C and each time for 5 min, and then PBS containing 2% collagenase was used to digest the tissues for 30 min at 37°C. After isolation, Dulbecco’s Modified Eagle Medium (DMEM; with 10% fetal bovine serum, 1% amphotericcin B, streptomycin, and penicillin) was used to culture the cells at 37°C with 5% CO_2_ and replaced the medium 2–3 days later. Anti-vimentin antibody staining was used to identify HVFs and selected the 4th- and 6th-generation cells for the next study [19,20].

### 2.3 Quantitative real-time polymerase chain reaction (qRT-PCR)

Trizol reagent was used to extract the total RNA from the HVFs or tissues isolated from normal controls or POP patients. RevertAid First Strand cDNA Synthesis kit (Thermo Fisher Scientific, Waltham, MA, USA) was used to reverse transcribe RNA and then used spectrophotometry to do the quantification. Applied Biosystems Prism 7300 (Applied Biosystems, Foster City, CA, USA) sequence detection system with Maxima SYBR Green/ROX qPCR Master Mix was used to perform the qRT-PCR following the manufacturer’s instructions. At last, GAPDH was used as the internal control. The relative expressions of IGF-1 and IGF-1 receptor (IGF-1R) were calculated using the 2^−ΔΔCq^ method.

### 2.4 Cell transfection

The control siRNA, IGF-1 siRNA, control plasmid, and IGF-1 plasmid were purchased from Invitrogen (Waltham, MA, USA) and transfected into HVFs and harvested the cells after 48 h for transfection. The manufacturer’s instructions were followed using the lipofectamine 3000 reagent (Life Technologies Corporation, Carlsbad, CA, USA) for cell transfection.

### 2.5 MTT assay

After certain treatment, HVFs were seeded at a density of 5,000 cells per well into 96 well plates and then the cell viability was determined. Briefly, 20 µL of MTT solution was added for each well and incubated at 37°C for 4 h, after that the medium was removed. Then 150 µL of dimethyl sulfoxide was added and the optical density was measured at 570 nm using multifunctional microplate reader (POLARstar OPTIMA; BMG, Offenburg, Germany).

### 2.6 Flow cytometry (FCM)

The cells from different groups were harvested and ice-cold PBS was used to wash them twice and then it was resuspended using 400 µL binding buffer. After that 5 µL fluorescein isothiocyanate-conjugated annexin V and 10 µL PI (Beyotime Institute of Biotechnology) was added to the buffer, incubated at room temperature in the dark for 20 min, and analyzed using FCM (BD LSR II; BD Biosciences, Franklin Lakes, NJ, USA). At last, the Flow Jo software 7.6 (BD Biosciences) was used to analyze the data.

### 2.7 Western blot assay

Protein expression was detected in the vaginal wall tissues of POP or HVFs using Western blot assay. The tissues were cut into small fragments and put in a glass homogenizer on ice then ground by hand, followed by the addition of 10 µL phenylmethanesulfonyl fluoride and 1,000 µL radio-immunoprecipitation assay (RIPA) lysis buffer (Wolsen, China). For cells, 1,000 µL RIPA lysis buffer (Wolsen, China) was added and incubated for 30 min on ice and then the proteins were separated using 12% sodium dodecyl sulfate–polyacrylamide gel and transferred to polyvinylidene fluoride membranes (Millipore, MA, USA) by electrophoresis. After that, the membranes were blocked in 5% skimmed milk at room temperature for 1.5 h. Then the membranes were exposed to the primary antibody at 4°C overnight. After washing thrice with phosphate buffer solution, i.e., Tween-20, the membranes were hybridized at room temperature for 2 h using horseradish peroxidase-conjugated anti-rabbit IgG secondary antibody. At last, enhanced chemiluminescence (Amersham Pharmacia, Piscataway, NJ, USA) was used to visualize the protein bands and ImageJ software [National Institutes of Health (NIH), Bethesda, MD, USA] was used to analyze the relative intensities of the protein bands.

### 2.8 Statistic analysis

All experiments were performed in triplicate and mean ± standard deviation was used to express the results. Then the data were analyzed using SPSS 18.0 software (Chicago, IL, USA). Student’s *t* test or one-way analysis of variance with Tukey’s *post hoc* test was used to perform the comparison between groups, and the *p* value <0.05 indicated statistical significance.

## 3 Results

### 3.1 IGF-1 expression was reduced in vaginal wall tissues from patients with POP

**Figure 1:**
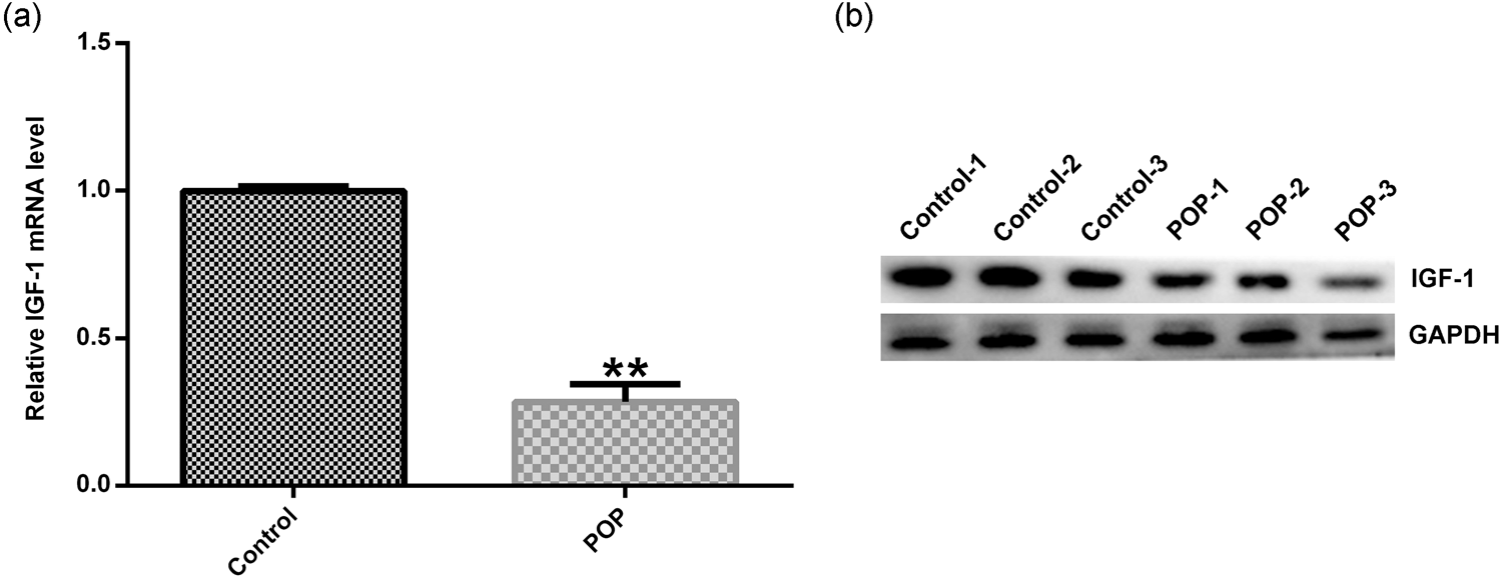
The expression of IGF-1 in the vaginal wall tissues of POP. Vaginal wall tissues were obtained from 30 patients having POP or other diseases that required hysterectomy and qRT-PCR (a) and Western blotting (b) were used to detect IGF-1 mRNA and protein expression. Data were reported as mean ± SD. **p < 0.01 vs control group.

The expression of IGF-1 in the vaginal wall tissues was compared between the normal subjects and patients with POP using the Western blot assay and qRT-PCR. The results showed that compared with the normal subjects, IGF-1 expression was significantly reduced at both mRNA and protein levels in the vaginal wall tissues obtained from patients with POP (Figure 1a and b).

### 3.2 IGF-1 siRNA treatment inhibited cell viability, promoted cell apoptosis, and downregulated the expression of Col I and Col III in HVFs

**Figure 2:**
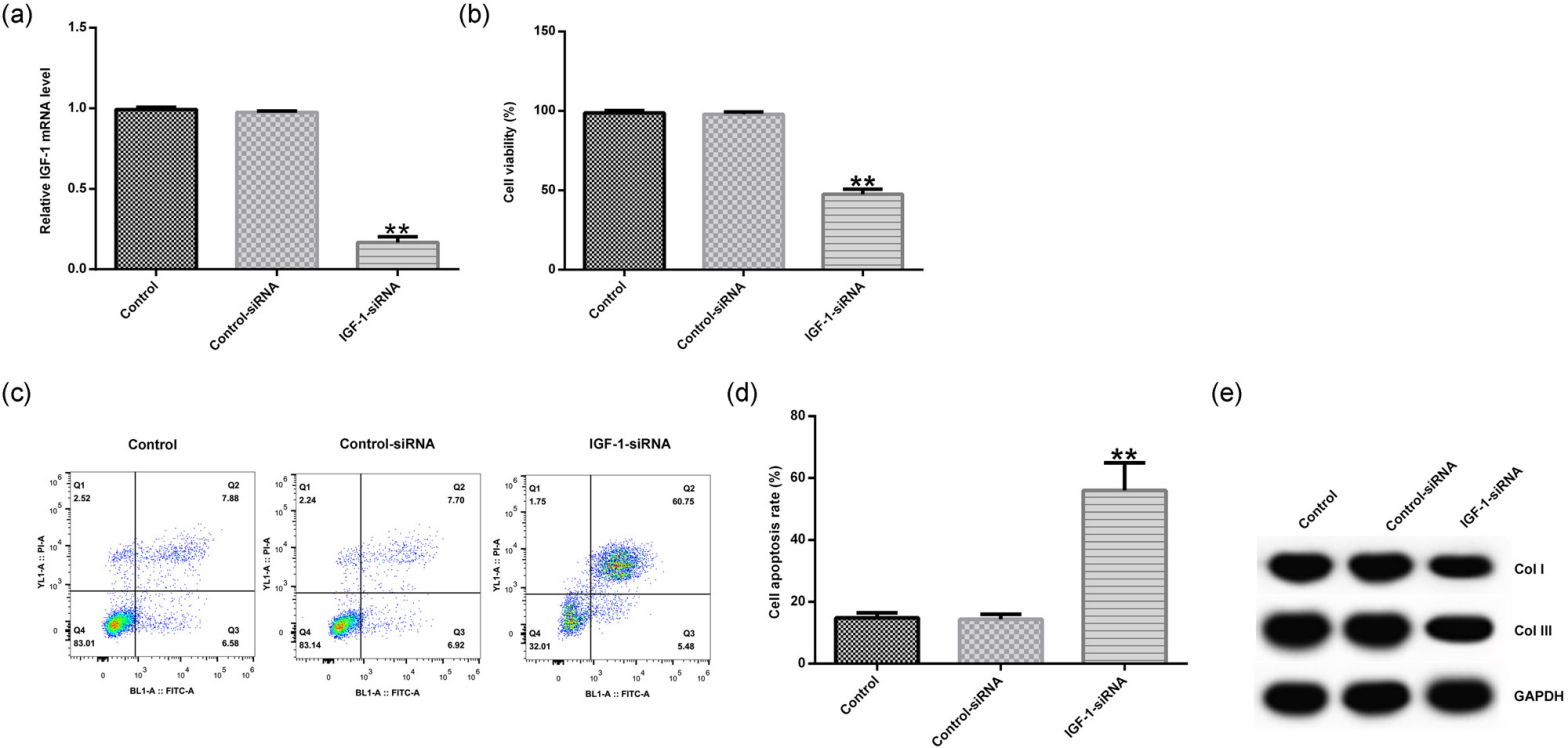
The effect of IGF-1 siRNA on the cell viability, cell apoptosis, and the protein expression of Col I and Col III in HVFs obtained from POP patients. Control siRNA and IGF-1 siRNA were transfected into HVFs and then MTT, FCM, and Western blotting assay were used to detect the cell viability, cell apoptosis, and the protein expression of Col I and Col III. (a) The effect of IGF-1 siRNA on IGF-1 mRNA expression in HVFs. (b) The effect of IGF-1 siRNA on the cell viability of HVFs. (c and d) The effect of IGF-1 siRNA on the cell apoptosis of HVFs. (e) The effect of IGF-1 siRNA on the protein expression of Col I and Col III in HVFs. Data were reported as mean ± SD. **p < 0.01 vs control group.

Considering the reduced expression of IGF-1 in POP, the role of IGF-1 in POP was further explored. The control siRNA and IGF-1 siRNA were transfected into HVFs. qRT-PCR showed that IGF-1-siRNA significantly inhibited IGF-1 mRNA expression in HVFs (Figure 2a). Then MTT analysis showed that knockdown of IGF-1 suppressed the cell viability of HVFs (Figure 2b). FCM analysis revealed that knockdown of IGF-1 induced the cell apoptosis of HVFs (Figure 2c and d). In addition, the expression of Col I and Col III was detected by Western blot analysis, and the results showed that knockdown of IGF-1 attenuated the expression of Col I and Col III in HVFs (Figure 2e).

### 3.3 IGF-1 plasmid treatment promoted cell viability, inhibited cell apoptosis, and upregulated the expression of Col I and Col III in HVFs

**Figure 3:**
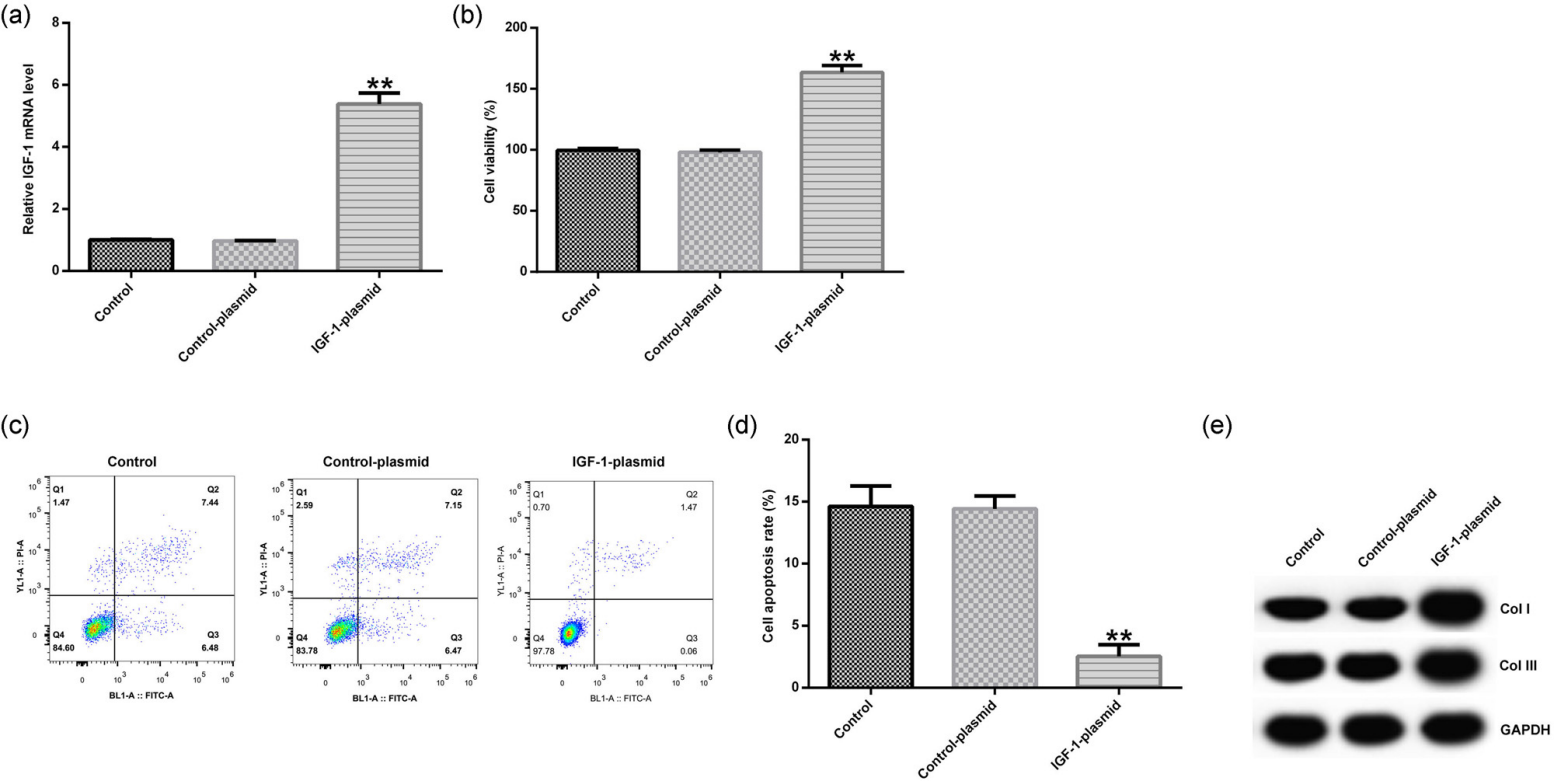
The effect of IGF-1 plasmid on the cell viability, cell apoptosis, and the protein expression of Col I and Col III in HVFs from POP patients. Control plasmid and IGF-1 plasmid were transfected into HVFs and then MTT, FCM, and Western blotting assay were used to detect the cell viability, cell apoptosis, and the protein expression of Col I and Col III. (a) The effect of IGF-1 plasmid on IGF-1 mRNA expression in HVFs. (b) The effect of IGF-1 plasmid on the cell viability of HVFs. (c and d) The effect of IGF-1 plasmid on the cell apoptosis of HVFs. (e) The effect of IGF-1 plasmid on the protein expression of Col I and Col III in HVFs. Data were reported as mean ± SD. **p < 0.01 vs control group.

In addition, the control plasmid and IGF-1 plasmid were transfected into HVFs. qRT-PCR showed that IGF-1 plasmid promoted the mRNA expression of IGF-1 in HVFs (Figure 3a). Then MTT analysis indicated that overexpression of IGF-1 increased the cell viability of HVFs (Figure 3b). FCM analysis revealed that overexpression of IGF-1 suppressed the cell apoptosis of HVFs (Figure 3c and d). In addition, the expression of Col I and Col III was detected by Western blot analysis, and the results showed that overexpression of IGF-1 promoted the expression of Col I and Col III in HVFs (Figure 3e).

### 3.4 IGF-1 affected the MAKP and NF-κB signaling pathways in HVFs

**Figure 4:**
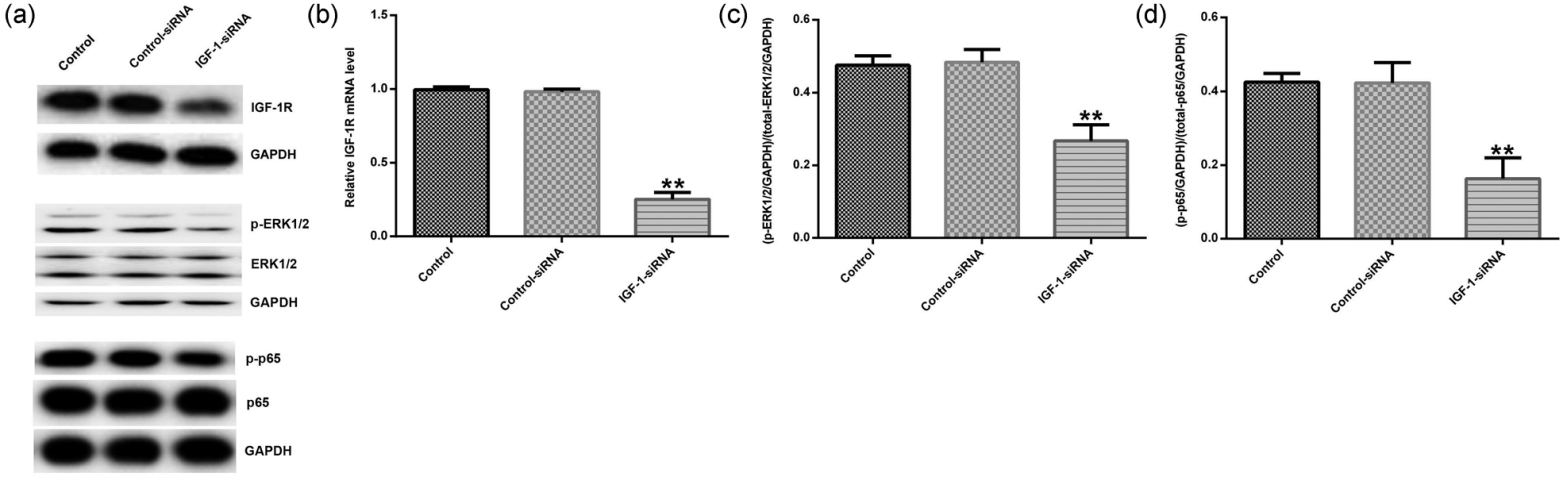
The effect of IGF-1 on MAKP and NF-κB signaling pathways in HVFs from POP patients. Control siRNA and IGF-1 siRNA were transfected into HVFs and then Western blotting assay was used to detect the protein levels of IGF-1R, p-ERK1/2, ERK1/2, p-p65 and p65; and qRT-PCR was used to measure the mRNA level of IGF-1R. (a) The effect of IGF-1 siRNA on IGF-1R, p-ERK1/2, ERK1/2, p-p65, and p65 protein expression in HVFs. (b) The effect of IGF-1 siRNA on IGF-1R mRNA expression in HVFs. (c and d) The effect of IGF-1 siRNA on p-ERK1/2/ERK1/2 and p-p65/p65 ratio. Data were reported as mean ± SD. **p < 0.01 vs control group.

**Figure 5:**
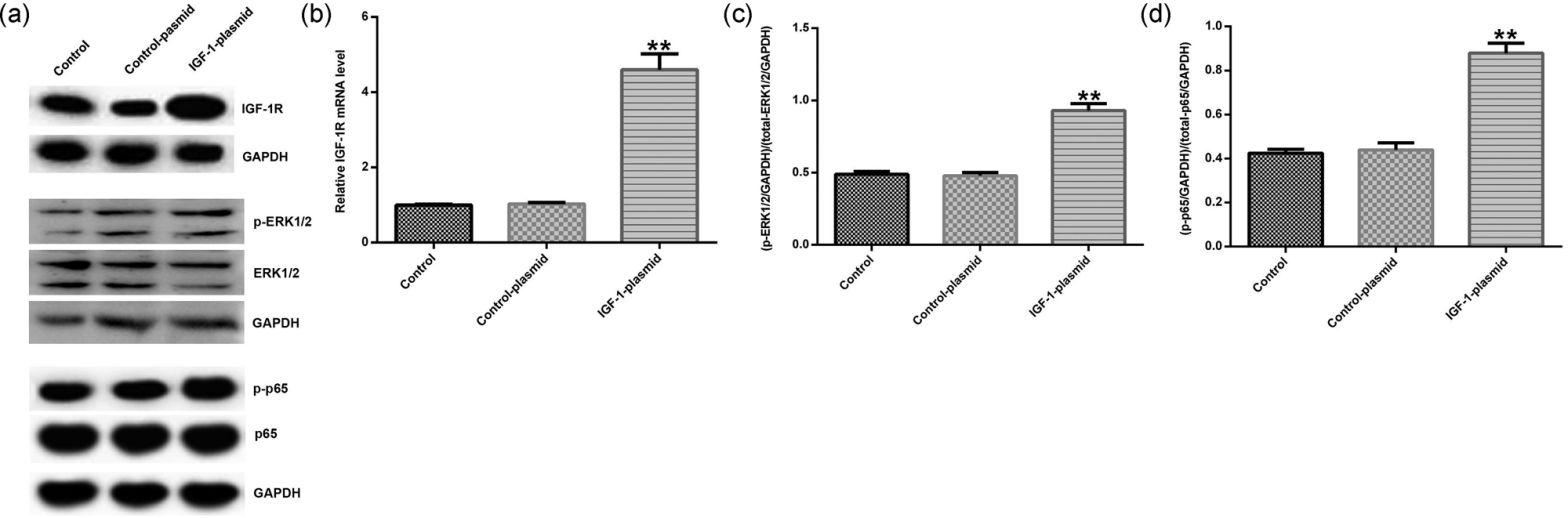
The effect of IGF-1 on MAKP and NF-κB signaling pathways in HVFs from POP patients. Control plasmid and IGF-1 plasmid were transfected into HVFs and then Western blotting assay was used to detect the protein level of IGF-1R, p-ERK1/2, ERK1/2, p-p65, and p65; and qRT-PCR was used to measure the mRNA level of IGF-1R. (a) The effect of IGF-1 siRNA on IGF-1R, p-ERK1/2, ERK1/2, p-p65, and p65 protein expression in HVFs. (b) The effect of IGF-1 siRNA on IGF-1R mRNA expression in HVFs. (c and d) The effect of IGF-1 siRNA on p-ERK1/2/ERK1/2 and p-p65/p65 ratio. Data were reported as mean ± SD. **p < 0.01 vs control group.

The control siRNA, IGF-1 siRNA, control plasmid, and IGF-1-plasmid were transfected into HVFs. Then Western blot assay was used to analyze the related protein expression of IGF-1R, MAKP, and NF-κB signaling pathways. The results showed that IGF-1 siRNA decreased the protein expression of IGF-1R, p-ERK1/2, and p-p65 (Figure 4a). IGF-1 siRNA also significantly decreased the mRNA expression of IGF-1R in HVFs (Figure 4b). Moreover, IGF-1 siRNA significantly decreased the ratio of p-ERK1/2/ERK1/2 (Figure 4c) and p-p65/p65 (Figure 4d) in HVFs. IGF-1 plasmid increased the protein expression of IGF-1R, p-ERK1/2, and p-p65 (Figure 5a), enhanced the mRNA level of IGF-1R (Figure 5b), and increased the ratio of p-ERK1/2/ERK1/2 (Figure 5c) and p-p65/p65 (Figure 5d) in HVFs.

## 4 Discussion

POP is a gynecological disease, which mainly consists of uterine prolapse and vaginal anterior or posterior wall bulging. POP is more common in older women, but its incidence in young women is also as high as 30% [21]. The current treatment of POP involves surgical treatment using anatomical recovery to achieve functional recovery. IGF-1 is closely related to cell proliferation, differentiation, survival, and maturation [15]. Therefore, in our study, the expression of IGF-1 in the vaginal wall tissues of POP patients was examined, and it was found that the expression of IGF-1 in the vaginal wall tissues of POP patients was significantly reduced. Then the mechanism of IGF-1 in POP was explored.

HVFs can affect the mechanical properties of the pelvic floor by controlling the integrity of collagen [22], so HVFs play important roles in the pathophysiology of POP. Primary culture of HVFs is commonly used to assess the connective tissues of POP. In this study, anti-vimentin antibody was used to identify fibroblasts isolated from vaginal tissues, and the results showed that the HVFs were successfully isolated. Amphotericin B was added to DMEM medium to avoid the effects of vaginal fungi, and this method was simple and efficient. Since this is a very basic and routine experiment, the results of anti-vimentin antibody staining were not retained. Anti-vimentin antibody staining results were not shown in this manuscript, and this was a limitation of this study.

Vaginal fibroblastic cells have been confirmed to play key roles in POP development [23]. Cell proliferation is a critical parameter in both normal and pathophysiological processes [24]. In order to explore the role of IGF-1 in POP, the effect of IGF-1 on the growth of HVFs was explored. The overexpressed IGF-1 was knocked down in HVFs obtained from POP patients, and it was found that the knockdown of IGF-1 inhibited the cell viability and promoted the cell apoptosis of HVFs. In contrast, overexpression of IGF-1 promoted the cell viability and inhibited the cell apoptosis of HVFs.

Considering that the reduction in Col I and Col III are the main causes of POP [7,8,9,10,11], the Western blot analysis was used to analyze the expression of Col I and Col III in HVFs, and it was found that the knockdown of IGF-1 reduced the expression of Col I and Col III, but overexpression of IGF-1 increased the expression of Col I and Col III in HVFs. This indicated that reduced expression of IGF-1 could promote the development of POP by inhibiting the expression of Col I and Col III.

It has been reported that IGF-l binds directly to IGF receptors on osteoblasts and directly inhibits the synthesis of collagenase in osteoclasts. It also promotes bone matrix synthesis and mineralization without relying on mitogenic action, affecting bone metabolism and promoting bone growth and development [25]. IGF-1R is the receptor for IGF-1 and includes two tyrosine kinase catalytic sites and one tyrosine kinase catalytic subunit according to its spatial structure. It has been reported that IGF1 binds to IGF-1R and regulates mitosis of cells, which is closely related to the development of tumors [26]. In this study, qRT-PCR and Western blot assay were used to detect IGF-1R expression, and it was found that IGF-1 siRNA significantly reduced IGF-1R mRNA and protein expression, and IGF-1-plasmid significantly promoted IGF-1R mRNA and protein expression in HVFs obtained from patients with POP.

Characterization of MAPK and NF-κB signaling pathway activation and downstream signaling was an important objective of this study. Decreased expression of Col I and Col III in POP vaginal tissues may be due to the involvement of MAPK and NF-κB signaling pathways in collagen synthesis and degradation [27]. As expected, our results showed that knockdown of IGF-1 inactivated MAPK and NF-κB pathways. Whereas overexpression of IGF-1 activated MAPK and NF-κB pathways in HVFs from patients with POP.

In conclusion, our results showed that IGF-1 could activate MAPK and NF-κB pathways and regulate collagen metabolism and the growth of vaginal wall fibroblasts, indicating the inhibitory effect of IGF-1 on the development of POP. Taken together, our study indicated that IGF-1 might be a new therapeutic target for POP.
